# Insights into the alteration of vaginal microbiota and metabolites in pregnant woman with preterm delivery: prospective cohort study

**DOI:** 10.3389/fcimb.2025.1580801

**Published:** 2025-09-18

**Authors:** Lihua Wang, Jun Zhang, Mengjun Zhang, Zhimin Xu, Yijing Zheng, Bingqing Lv, Mian Pan

**Affiliations:** Department of Obstetrics & Gynecology, Fujian Maternity and Child Health Hospital, College of Clinical Medicine for Obstetrics & Gynecology and Pediatrics, Fujian Medical University, Fuzhou, China

**Keywords:** preterm birth, vaginal microbiome, vaginal metabolomics, microbial-marker, metabolic pathway

## Abstract

Disruptions in vaginal microbiota and metabolites during pregnancy may be the most important risk factor for preterm delivery, thus the difference in vaginal microbiota and metabolites between women who subsequently delivered at term and who eventually experienced preterm birth. In this study, 63 participants were enrolled before the cervical cerclage surgery (namely pre-cerclage), comprising women who subsequently delivered at term and who eventually experienced preterm birth. The cervical-vaginal fluid (CVF) was collected two days prior to the cervical cerclage surgery. Compared with the term birth groups (PrTG), the proportion of beneficial bacteria (*Lactobacillus*, *Prevotella*, *Trichococcus*, *Neisseria* and *Gemella*) in the preterm birth group (PrPG) were significantly reduced (*p* < 0.05), while the proportion of harmful bacteria (*Thauera*, *Ochrobactrum*, *Gardnerella*, *Massilia*, *Phyllobacteriaceae* and *Atopobium*) were significantly increased (*p* < 0.05). In addition, vaginal metabolomics-based LC-Orbitrap-MS/MS revealed that the contents of 2-Piperidone, Melphalan, N-acetylputrescine, Obatoclax, Eurostoside, Pregnanediol 3-O-glucuronide, O-Phospho-L-serine, 1-Kestose and N-arachidonylglycine were significantly decreased in the PrPG group compared with the PrTG group, while Acenocoumarol, Isopyrazam, Pentosidine, hexose, 7-Hydroxymitragynine, PE, Tamoxifen and 1-Deoxynojirimycin contents were significantly increased. These results suggest that specific bacterial species and metabolites may serve as potential biomarkers for preterm birth prediction, and approve the theoretical basis for the intervention of preterm birth.

## 1 Introduction

Preterm delivery is described as birth occurring before 37 weeks of gestation (or less than 259 days from the first day of a woman’s last menstrual period), which includes spontaneous and iatrogenic preterm births. Preterm delivery is the major cause of infant morbidity and mortality worldwide and is related to long-time adverse outcomes in children (Xie et al., 2022). The high incidence of preterm deliveries not only imposes a significant annual economic burden on society, but also undermines family well-being and social harmony. According to the Global Disease Burden Study from 2022, over 15 million babies are born preterm annually, and the prevalence of preterm birth is on the rise globally (Adane et al., 2024). Among these preterm births, approximately 45% are diagnosed with spontaneous preterm labor with intact membranes, and approximately 30% of premature infants are diagnosed with spontaneous preterm labor with ruptured membranes in the world (Beernink et al., 2023). According a previous report, the occurrence of preterm delivery is associated with factors, such as a short cervix, extremes of maternal age (< 25 years and > 35 years) and body mass index (BMI, from 18 to 28), low socio-economic status, smoking, and genetic polymorphisms (Wei et al., 2024). There is obvious difference in the incidence rate of preterm delivery among different regions. For example, it is reported that the rates of preterm delivery accounted for more than 80% of global cases in low-income and middle-income countries, such as Southern Asia and Sub-Saharan Africa (Ohuma et al., 2023). At present, cervical cerclage and vaginal progesterone are widely used in the preventive treatment of preterm delivery and preterm premature rupture of membranes. Cervical cerclage mechanically maintains a long and closed cervix, which is one of the universal methods applied to decrease preterm birth in pregnant women with older age or high risk (Seyama et al., 2022). However, there is an obvious difference in the intervention effect of cervical cerclage among the different people.

As is well known, the vaginal microbiota is an essential regulator of reproductive tract pathophysiology, but the diversity of vaginal microbiome was obviously lower than that of other mucosal surfaces (Golob et al., 2024). A previous study indicated that pregnant women diagnosed with bacterial vaginosis-associated vaginal dysbiosis before 20 weeks of gestation face a five-fold increased risk of late miscarriage or preterm birth before 34 weeks, with the risk rising to sevenfold when bacterial vaginosis is detected before 16 weeks (Peelen et al., 2019). *Lactobacillus* species are the major bacterium in the vagina, and are generally regarded as a hallmark of health, especially during reproductive years. A high proportion of *Lactobacillus* in the vagina is beneficial for elevating the level of short-chain fatty acids that provide energy for the growth of vaginal epithelial cells and suppress the growth of harmful bacteria (Hong et al., 2021). Another study displayed that elevating the abundance of *Lactobacillus* effectively suppressed the production of pro-inflammatory cytokines, relieves oxidative stress, and regulated the composition of vaginal microbiota (Chee et al., 2020). On the contrary, the reduction of *Lactobacillus* species and increases in microbial diversity elevate the risk of bacterial vaginosis, which may be associated with the high rate of preterm delivery (Fettweis et al., 2019). The changes in vaginal microbiota induce the alterations in vaginal metabolite contents. Microbial metabolites are reported to have prominence and various influences on human health and are detectable in a series of biological tissues, including the colon, liver, brain and vagina (Guo et al., 2023a, 2023). At present, nuclear magnetic resonance (NMR), liquid chromatography tandem mass spectrometry (LC-MS) and gas chromatography-mass spectrometry (GC-MS) are extensively applied to detect the metabolites because of their relatively high sensitivity, which is beneficial for distinguishing different vaginal metabolites between the pregnant woman who later experienced preterm delivery and term birth.

The association between the vaginal microbiota and the occurrence of preterm delivery is widely investigated, which may not accurately predict premature birth. In the present study, the vagina microbiota and vaginal metabolites between pregnant women (years: 21–38 and BMI: 19.57-28.00) who later experienced preterm delivery and term birth from Fujian maternity and child health hospital (Fuzhou, China) before the cervical cerclage surgery (namely pre-cerclage) were measured by 16S rRNA gene sequencing and untargeted metabolomics, respectively. Then, the key microbial phylotypes and marked metabolites in the vagina of pregnant women who eventually experienced preterm delivery were screened using statistical analysis, which offers useful information to predict preterm delivery or the development of new therapeutics for pregnant women who later experienced preterm delivery.

## 2 Materials and methods

### 2.1 Participant recruitment

In this study, participants were recruited from Fujian maternity and child health hospital (Fuzhou, China) between 2021 and 2023, and were supported by the Ethics Committee of Fujian Maternity and Child Health Hospital (Approval No. 2021KLR601). A total of 132 participants, who had not used antibiotics, engaged in sexual activity, or used tobacco in the 12 weeks prior, provided written informed consent before enrollment in accordance with the approved institutional guidelines. However, only 63 participants met the experimental requirements (excluding 46 cases of multiple pregnancies, 20 cases of lost contact, 2 cases of uterine malformations, and 1 case of severe fetal malformations).

### 2.2 Data collection

The current and historical pregnancy outcomes of participants were collected and recorded, including maternal age, body mass index (BMI), abortion frequency and fertility frequency). Before the cervical cerclage surgery (namely pre-cerclage), all participants were divided into 2 groups according to the outcome of pregnancy, namely the preterm delivery group (PrPG) and the term birth groups (PrTG).

### 2.3 Sample collection

A single-use sterile endoscope was used to expose the vagina, and then a cotton swab was gently rotated across the vaginal wall for 20 s. The sampling loop was taken out from the cannula to fully exposed in the uterine, then rotate the handle of the sampler ten times to collect the endometrial sample, and hen rotate the handle of the sampler ten times to collect the endometrial sample. In the passage of the sampler through the vagina, the sampling loop remains retracted within the cannula to avoid contact with microorganisms from these two body parts, thus eliminating cross-contamination between intrauterine and vaginal samples. All samples were immediately frozen using liquid nitrogen for 3–4 min, and then placed in -80 °C until further use. To avoid potential interference from the cervical cerclage surgery on vaginal microbiota and their metabolites, the cervical-vaginal fluid (CVF) was collected two days prior to surgery. In addition, the clinical patients enrolled in this study subsequently underwent surgical treatment following sample collection. The specific surgical steps and preoperative management of cervical cerclage surgery were carried out according to our previous study (Fang et al., 2020).

### 2.4 16S rRNA gene sequencing

The sequencing analysis of vaginal microbiota was implemented by the MiSeq platform based on the methodology outlined in a previous report with slight modifications (Guo et al., 2025a). In brief, total bacterial DNA from CVF sample was extracted using a FastDNA™ Fecal Kit (MoBio, Carlsbad, CA, USA), and then the V3-V4 regions of bacterial 16S rRNA genes were amplified by broad-range bacterial primers, namely 338F primers (5′-CCTAYGGGRBGCASCAG-3′) and 806R primers (5′-GGACTACHVGGGTWTCTAAT-3′). These products were purified using 2.0% agarose gel electrophoresis, target fragment was collected and recovered by a QIAquick Gel Extraction kit (BeckmanA63880, Hangzhou, China). Sequencing libraries consisted of equal concentrations of each sample, and their quality was evaluated on the Qubit@ 2.0 Fluorometer (Thermo Fisher Scientific, CA, USA), and then was implemented on Illumina Miseq platform (San Diego, CA, USA) at Shanghai Biotree Biotech. Co., Ltd.

The raw data were filtered, denoised, merged and chimera removed using Microbial Ecology software (v 2.0), and the high-quality sequences were collected, and then grouped into operational taxonomic units (OTUs) with similarities more than 97%. Taxonomy annotation process was carried out on the OTU sequences by the Mothur approach and the SSU rRNA database of SILVA138.1. Alpha and beta diversity of vaginal microbiota were analyzed by X shell (v 7.0). The overall differences of vaginal microbiota were assessed based on the principal coordinates analysis (PCoA, based on Bray method) by R software (v 4.4.2), the key microbial phylotypes were screened using Microbial Ecology software.

### 2.5 Untargeted metabolomics analysis

Untargeted metabolomics analysis was implemented by liquid chromatography-Orbitrap-mass spectrometry/mass spectrometry (LC-Orbitrap-MS/MS, Thermo Fisher Scientific, CA, USA) based on a previous report with minor modifications (Guo et al., 2025). Briefly, the vaginal contents were freeze-dried, weighted, and extracted using the organic solution (methanol: acetonitrile = 1:1). The mixture solution was sufficiently vibrated using a high-throughput oscillator, and placed at 0-4°C environment. After 2 h of stillness, the supernatant of each sample was collected by centrifugation (12000 g, 10 min, 2°C), and then dried at 25°C under a vacuum environment. The sediment of each sample was resuspended using the organic solution (methanol: acetonitrile = 1:1), the supernatant was collected by centrifugation (12000 g, 15 min, 2°C), and then filtrated by 0.22-μm aqueous membrane. Quality control samples consist of an equal volume of each sample, so as to assess the stability of instruments during the experiment.

The vagina metabolic profiling was analyzed using LC-Orbitrap-MS/MS with an ACQUITY UPLC BEH Amide column (50 × 2.1 mm, 1.7 µm; Waters, Milford, USA). Among these, MS detection of metabolites was carried out on Orbitrap Exploris 120 (Thermo Fisher Scientific, CA, USA) with an ESI ion source in positive and negative modes. Mobile phase A contained 0.1% formic acid and 5 mM ammonium acetate, and the mobile phase B was acetonitrile. The raw data were preliminarily treated using ProteoWizard software and R software (v 4.2.2), including peak alignment, peak identification, and deconvolution. Principal components analysis (PCA, based on Z-score normalization method), partial least squares discriminant analysis (PLS-DA, based on Kernelpls method), and orthogonal partial least-squares discrimination analysis (OPLS-DA) of vaginal metabolomics were carried out by MetaboAnalyst (v 6.0). Differential vaginal metabolites between the PrPG and PrTG groups were screened by S-loading plot based on OPLS-DA. The proportion of obviously different vaginal metabolites (VIP > 1.0, and *p* < 0.05) between the PrPG and PrTG groups was analyzed by R software (v 4.4.2).

### 2.6 Statistical analysis

All data of the present study were presented as the mean ± SD using GraphPad Prism 9.0 (GraphPad Software, Santiago, USA). The significant differences between the two groups were assessed based on Unpaired Student’s t test or Mann-Whitney U test using SPSS 24.0 statistical package (IBM Corporation, Armonk, NY, USA).

## 3 Results

### 3.1 Clinical characteristics and pregnancy outcome of participants

As shown in **Table 1**, a total of 63 participants were recruited in this study, including 32 women (50.79%) who delivered at term birth and 31 women (49.21%) who later experienced preterm delivery. No significant difference was observed between the PrPG and PrTG groups in maternal age, BMI, gravida, parity and cervical length (*p* > 0.05), but gestational age at delivery and birth weight in the PrTG group were higher than that in the PrPG group (*p* < 0.05).

**Table 1:** Descriptive statistics of study participants. Data are presented as mean ± SD.

Characteristic	PrPG (n =32)	PrTG (n =31)	*P* Value
Age (years)	31.19 ± 3.68	30.65 ± 3.58	0.56
BMI (m^2^/kg)	23.85 ± 3.54	22.79 ± 3.34	0.22
Gravida	3.0 ± 1.44	2.29 ± 1.01	0.057
GA at the time point of sampling	22.25 ± 2.68	22.45 ± 2.66	0.77
Parity	0.56 ± 0.67	0.42 ± 0.56	0.36
Cervical length (cm)	1.34 ± 0.67	1.70 ± 1.17	0.24
GA at delivery (weeks)	32.27 ± 3.29	38.16 ± 1.03	5.90×10^-11^
Birth weight (g)	2007.93 ± 559.58	3188.07 ± 435.89	3.50×10^-12^
Able to manage on income available	Not too bad	Not too bad	–

BMI, body mass index; GA, Gestational age. All participants (from China) had no history of preterm birth (PTB), miscarriage, fetal loss, substance use (smoking, drugs, or alcohol). Progesterone was administered to all participants without cervical cerclage indications during the perioperative period (within one week before and after surgery).

### 3.2 Change of vaginal microbiota in pregnant women who eventually experienced preterm delivery

**Figure 1:**
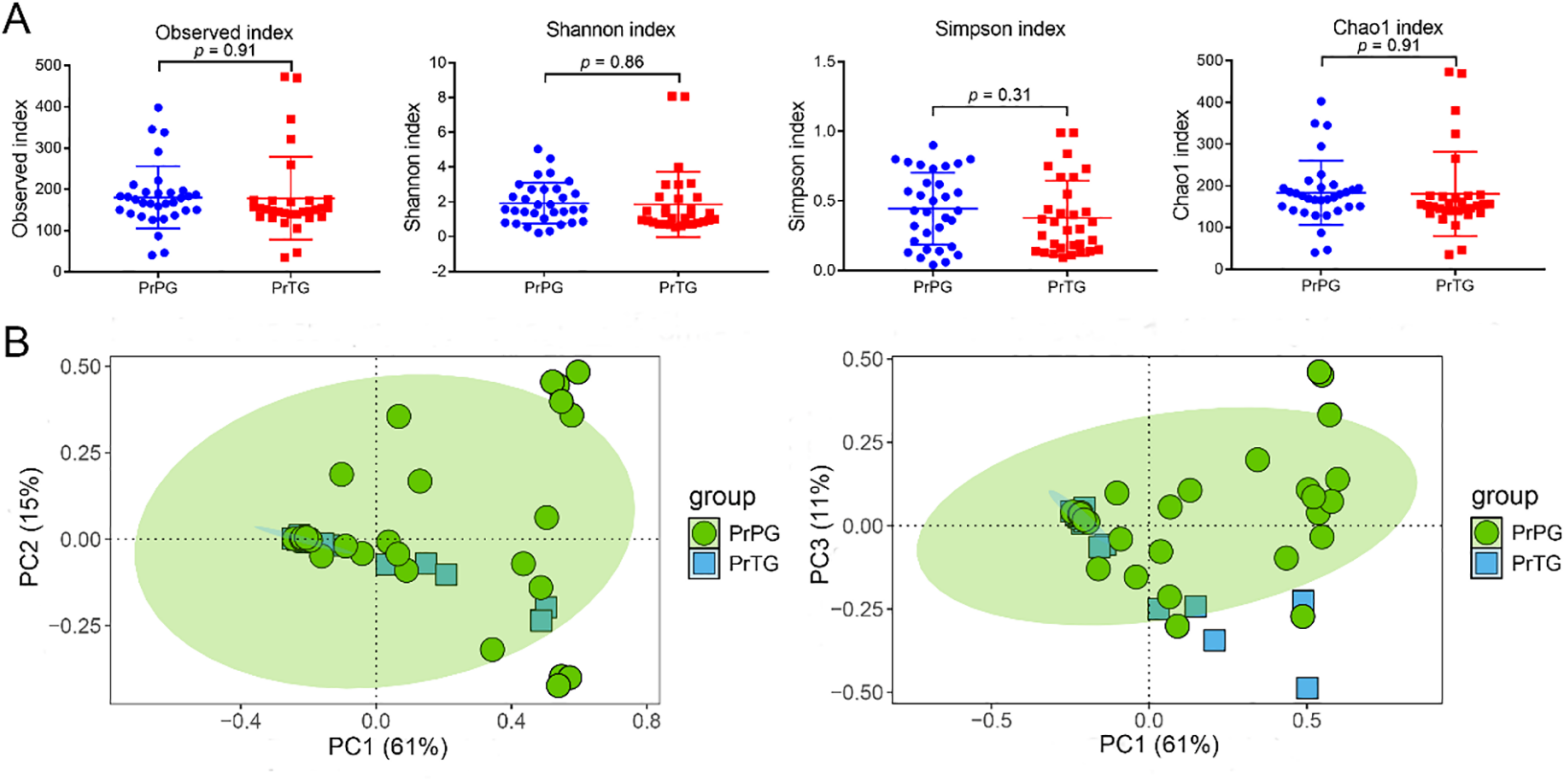
The diversity of between pregnant women who later experienced preterm delivery and term birth. **(A)** Observed, Shannon, Simpson and Chao1 indexes, and **(B)** PCoA analysis based on Bray-Curtis distance.

The vaginal microbiome diversity and composition were analyzed by 16S rDNA sequencing. As indicated in **Figure 1A**, the observed (180.53 ± 75.71 vs 178.10 ± 100.50, *p* = 0.91), Shannon (1.93 ± 1.19 vs 1.86 ± 1.87, *p* = 0.86), Simpson (0.45 ± 0.26 vs 0.38 ± 0.27, *p* = 0.31) and Chao1 (183.19 ± 76.81 vs 180.59 ± 101.12, *p* = 0.91) indexes of vaginal microbiota in the PrPG group were slightly higher than that in the PrTG group (*p* > 0.05). Among these, Shannon, Simpson, and Chao1 indexes are widely used to evaluate overall community complexity, emphasize dominant species, and address rare taxa to avoid underestimation of diversity, respectively. The Venn diagram showed that the quantity of operational taxonomic units (OTUs) shared by the two groups was 839, with the number of unique OTUs in the PrPG and PrTG groups being 1470 and 1301 respectively (**Supplementary Figure S1**). In addition, principal coordinates analysis (PCoA) was used to investigate the association between preterm delivery and vaginal microbiota composition (**Figure 1B**). The first principal components (PC1), second principal components (PC2) and third principal components (PC3) contributed 61%, 15% and 11% of the total variance in the PCoA score plot, respectively. Samples from the PrTG group were predominantly distributed along the negative of PC1, whereas those from the PrPG group exhibited a more dispersed distribution, suggesting that the disorder of vaginal microbiota was presented in pregnant women who later experienced preterm delivery.

### 3.3 Screening for key microbial phylotypes

**Figure 2:**
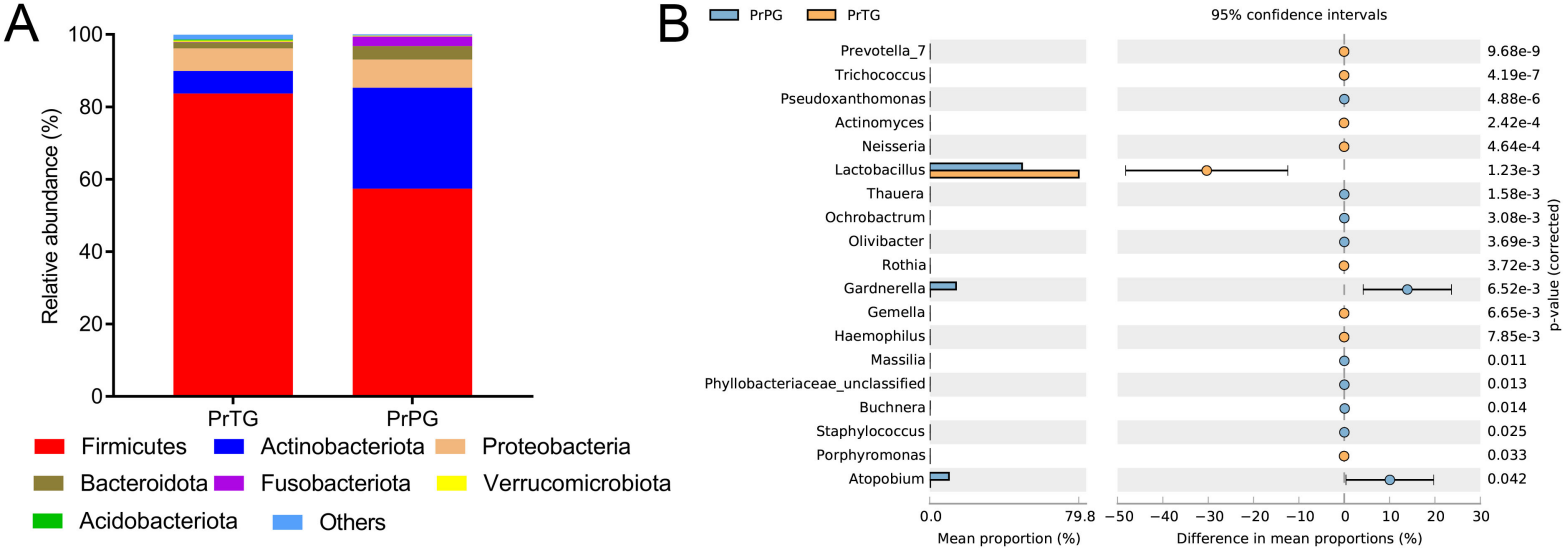
The relative abundance of vaginal microbiota between pregnant women who later experienced preterm delivery and term birth before cervical cerclage. **(A)** phylum and **(B)** genus.

The changes in microbiota composition in the vagina from pregnant women who later experienced preterm delivery and term birth were revealed at differing levels. Firmicutes, Actinobacteriota, Proteobacteria, Bacteroidota, Fusobacteriota, Verrucomicrobiota and Acidobacteriota were mainly microorganisms in the PrPG and PrTG groups at the phylum level (**Figure 2A**). Notably, compared with the PrTG group, the proportion of Firmicutes (from 83.68% to 57.42%), Verrucomicrobiota (from 0.39% to 0.20%) and Acidobacteriota (from 1.35% to 0.31%) in the PrPG group was obviously reduced (*p* < 0.05), but the proportion of Actinobacteriota (from 6.23% to 27.92%), Proteobacteria (from 6.24% to 7.78%), Bacteroidota (from 1.69% to 3.70%) and Fusobacteriota (from 0.11% to 2.67%) was remarkably increased (*p* < 0.05). At the genus level, the proportion of *Prevotella*, *Trichococcus*, *Actinomyces*, *Neisseria*, *Lactobacillus*, *Rothia*, *Gemella*, *Haemophilus* and *Porphyromonas* was remarkably reduced in the PrPG group compared with that in the PrTG group (*p* < 0.05), but the relative abundance of *Pseudoxanthomonas*, *Thauera*, *Ochrobactrum*, *Olivibacter*, *Gardnerella*, *Massilia*, *Phyllobacteriaceae*_*unclassified*, *Buchnera*, *Staphylococcus* and *Atopobium* was significantly increased (*p* < 0.05) (**Figure 2B**).

### 3.4 Change of vagina metabolic profiling in pregnant women who later experienced preterm delivery

**Figure 3:**
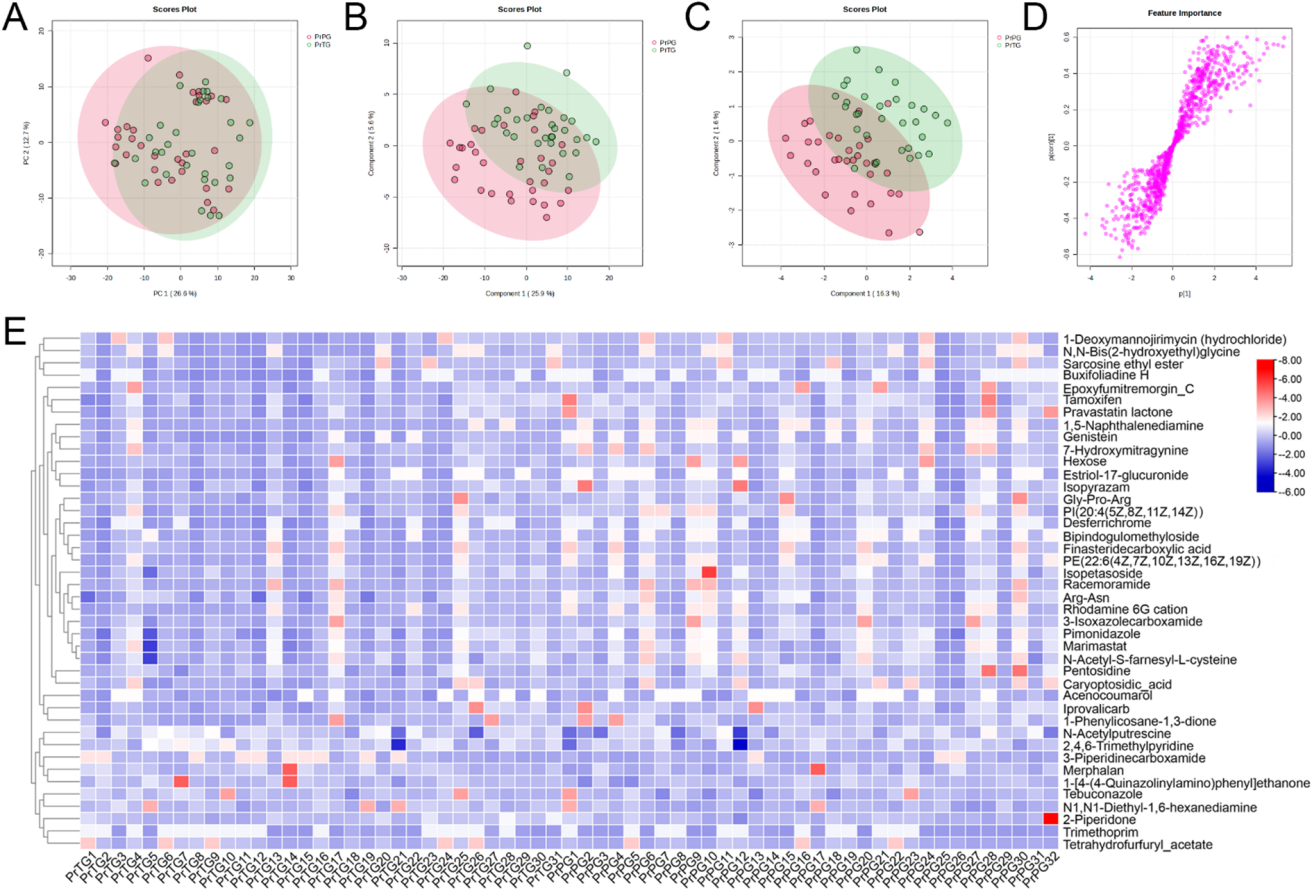
The alteration in vaginal metabolites in pregnant women who later experienced preterm delivery in the positive ion modes. **(A)** PCA, **(B)** PLS-DA, **(C)** OPLS-DA, **(D)** S-plots of OPLS-DA, and **(E)** Heatmap exhibited the differential metabolites between the PrPG and PrTG groups.

**Figure 4:**
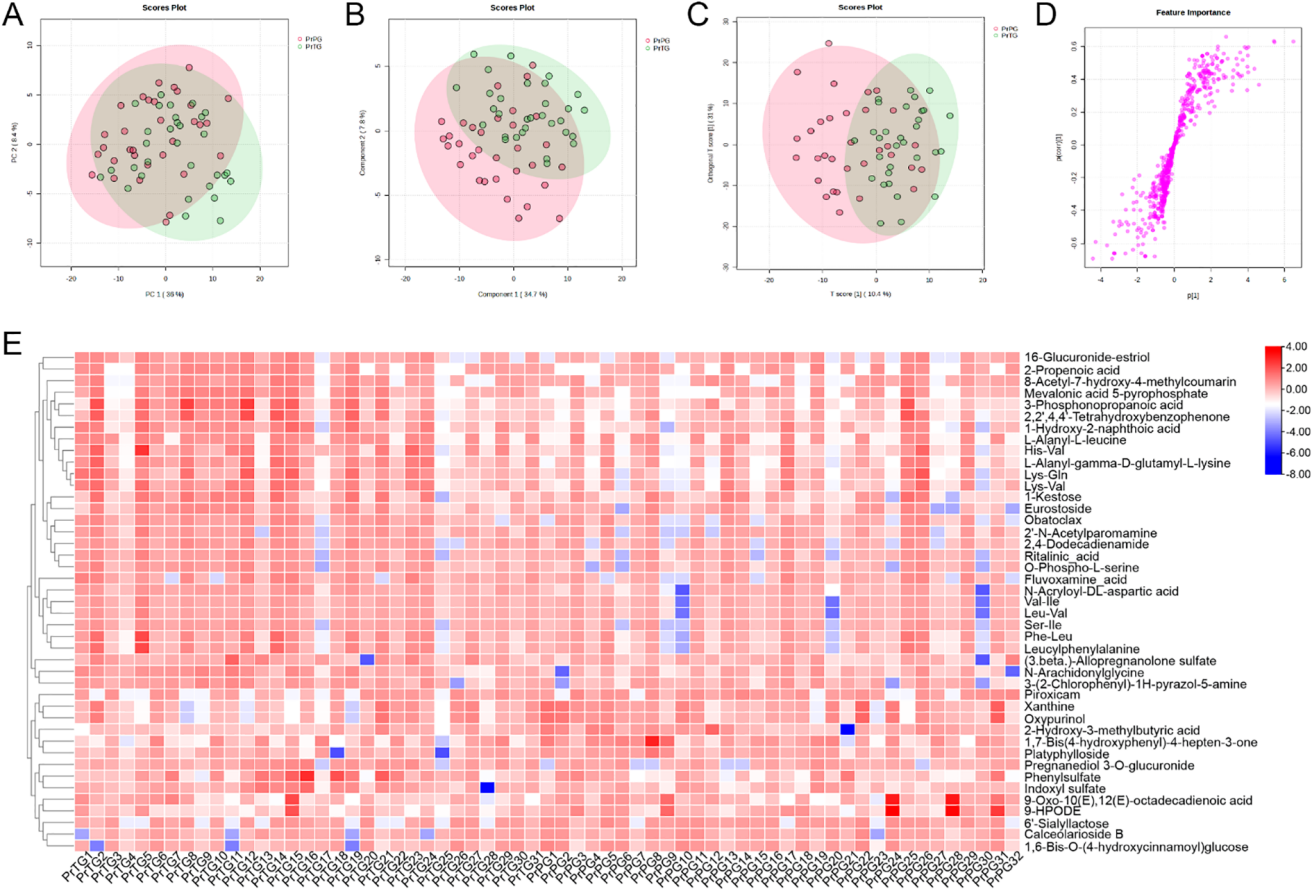
The alteration in vaginal metabolites in pregnant women who later experienced preterm delivery in the negative ion modes. **(A)** PCA, **(B)** PLS-DA, **(C)** OPLS-DA, **(D)** S-plots of OPLS-DA, and **(E)** Heatmap exhibited the differential metabolites between the PrPG and PrTG groups.

In the present study, vaginal metabolites between the PrTG and PrPG groups were detected by untargeted metabolomics based on LC-Orbitrap-MS/MS. PCA was used to reveal metabolic profile clustering between pregnant women who later experienced preterm delivery and term birth. The result of PCA analysis displayed that PC1 and PC2 contributed for 26.6% and 12.7% of the total variation in the positive ion modes (**Figure 3A**), while PC1 and PC2 accounted for 36.0% and 8.4% of the total variation in the negative ion modes (**Figure 4A**). A clear separation was observed between the PrTG and PrPG groups, suggesting that alteration of vagina metabolic profiling may be one of the essential causes for preterm delivery. Subsequently, PLS-DA and OPLS-DA were applied to further revealed the changes in vagina metabolites (**Figures 3B, C**, **4B, C**). Partial least squares discriminant analysis (PLS-DA) and orthogonal partial least-squares discrimination analysis (OPLS-DA) scores plot showed an obvious distinction between the PrTG and PrPG groups in both the positive and negative ion modes. Furthermore, the S-plots of OPLS-DA confirmed differences in the vaginal metabolites between the PrTG and PrPG groups (**Figures 3D**, **4D**).

### 3.5 Screening for differential vagina metabolites

Based on S-plot analysis of the OPLS-DA model, a total of 42 differential metabolites were identified between the PrTG and PrPG groups in positive ion mode (VIP value > 1 and *p* < 0.05) (**Figure 3E**). Among them, the levels of 2,4,6-Trimethylpyridine, N1,N1-Diethyl-1,6-hexanediamine, Tebuconazole, 1-[4-(4-Quinazolinylamino)phenyl]ethenone, Trimethoprim, Merphalan, 3-Piperidinecarboxamide, Tetrahydrofurfuryl_acetate, 2-Piperidone and N-Acetylputrescine in the PrPG group were significantly reduced compared with that in the PrTG group, while the levels of Pimonidazole, 1,5-Naphthalenediamine, Racemoramide, Acenocoumarol, Caryoptosidic_acid, Finasteridecarboxylic acid, Iprovalicarb, Gly-Pro-Arg, Isopyrazam, Pravastatin lactone, Pentosidine, 1-Phenylicosane-1,3-dione, Epoxyfumitremorgin_C, 3-Isoxazolecarboxamide, Bipindogulomethyloside, Marimastat, Hexose, Arg-Asn, Buxifoliadine H, 7-Hydroxymitragynine, Estriol-17-glucuronide, PE(22:6(4Z,7Z,10Z,13Z,16Z,19Z)), Isopetasoside, Rhodamine 6G cation, Desferrichrome, Tamoxifen, 1-Deoxymannojirimycin (hydrochloride), N-Acetyl-S-farnesyl-L-cysteine, N,N-Bis(2-hydroxyethyl)glycine, PI(20:4(5Z,8Z,11Z,14Z)), Genistein and Sarcosine ethyl ester were significantly increased.

In the negative ion modes, a total of 43 differential metabolites between the PrTG and PrPG groups were screened (VIP value > 1 and *p* < 0.05) (**Figure 4E**). Among these, the proportion of 2-Hydroxy-3-methylbutyric acid, 1,7-Bis(4-hydroxyphenyl)-4-hepten-3-one, Platyphylloside, Piroxicam, Oxypurinol, Xanthine, 1,6-Bis-O-(4-hydroxycinnamoyl)glucose, 6’-Sialyllactose, 9-Oxo-10(E),12(E)-octadecadienoic acid, Calceolarioside B and 9-HPODE in the PrPG group was significantly elevated compared with the PrPG group, but the proportion of Lys-Val, Obatoclax, L-Alanyl-gamma-D-glutamyl-L-lysine, Ser-Ile, L-Alanyl-L-leucine, Val-Ile, Leu-Val, Lys-Gln, Eurostoside, 2’-N-Acetylparomamine, Pregnanediol 3-O-glucuronide, Ritalinic_acid, 1-Hydroxy-2-naphthoic acid, Leucylphenylalanine, Phe-Leu, N-Acryloyl-DL-aspartic acid, O-Phospho-L-serine, 3-(2-Chlorophenyl)-1H-pyrazol-5-amine, 16-Glucuronide-estriol, 2,2’,4,4’-Tetrahydroxybenzophenone, Fluvoxamine_acid, 1-Kestose, Indoxyl sulfate, 2-Propenoic acid, His-Val, 2,4-Dodecadienamide, N-Arachidonylglycine, (3.beta.)-Allopregnanolone sulfate, Phenylsulfate, 3-Phosphonopropanoic acid, 8-Acetyl-7-hydroxy-4-methylcoumarin and Mevalonic acid 5-pyrophosphate was significantly decreased.

### 3.6 Metabolic pathway analysis

**Figure 5:**
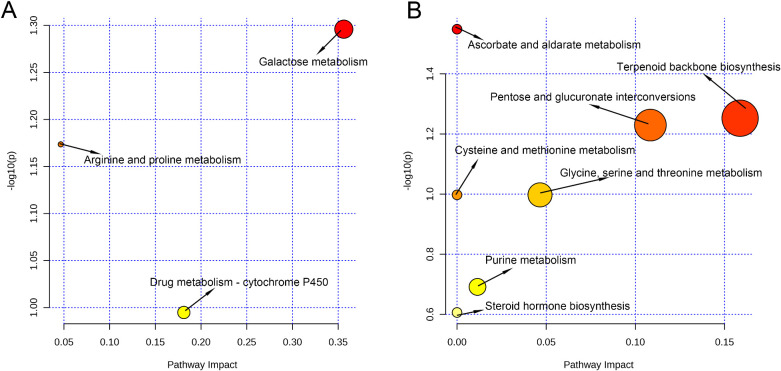
Analysis of key metabolic pathways (each point represents a metabolic pathway). **(A)** Pathway analysis in the positive ion modes and **(B)** in the negative ion modes. The *p* values calculated from the enrichment analysis are indicated by a color gradient: from white (highest *p* value) to red (lowest *p* value), while the pathway impact value calculated from pathway topology analysis is indicated by dot size.

Untargeted metabolomic profiling revealed significant differences in vaginal metabolites between PrTG and PrPG groups. These differential metabolites were analyzed using MetaboAnalyst 6.0 to identify associated metabolic pathways through the KEGG database. In the metabolic pathway, each circle expresses one metabolic pathway, and the size and color of the circles depend on the importance and *p*-values of the pathway. In the positive ion modes, galactose metabolism, arginine and proline metabolism, and drug metabolism-cytochrome P450 were significantly disturbed in pregnant women who later experienced preterm delivery (*p* < 0.05, **Figure 5A**). In the negative ion modes, ascorbate and aldarate metabolism, terpenoid backbone biosynthesis, pentose and glucuronate interconversions, cysteine and methionine metabolism, glycine, serine and threonine metabolism, purine metabolism and steroid hormone biosynthesis were significantly disturbed in the pregnant women who later experienced preterm delivery (*p* < 0.05, **Figure 5B**). These results revealed that the mentioned metabolic pathways were associated with preterm delivery in pregnant women.

## 4 Discussion

Preterm delivery has become an alarming public health concern due to the relatively higher mortality rate presented in preterm infants. According to the previous report, approximately three-quarters of preterm delivery cases are diagnosed as spontaneous preterm delivery, which includes previous spontaneous preterm delivery or preterm pre-labor rupture of the membranes (Humberg et al., 2020). Although the great progress made in cervical cerclage, there are substantial differences in treatment effectiveness among different populations (Park et al., 2022). Gut microbiota and its metabolites play a vital role in enhancing the host’s health, such as exhibiting hypoglycemic, hypolipidemic, antidiabetic, and antidepressant effects. Therefore, we hypothesized that preterm delivery is related to the alterations in vaginal microbiota and its metabolites. In the present study, 16S rRNA gene sequencing and untargeted metabolomics were used to detect and identify the vaginal microbiota and its metabolites, with the aim of screening the key microbial phylotypes and differential vagina metabolites.

The risk of preterm delivery is strongly related to the BMI of pregnant women. Specifically, a BMI above 28.0 or below 18.0 increases the risk of preterm delivery (Cnattingius et al., 2013). A high BMI in pregnant women elevates the likelihood of gestational diabetes, hypertensive disorders and fetal malformations, which are among the most significant factors contributing to medically indicated preterm delivery (Robinson et al., 2021). In addition, advanced maternal age (more than 35 years) and young maternal age (less than 25 years) also elevate the risk of preterm delivery (Wu et al., 2024). Therefore, participants were collected based on a BMI within the range of 18 to 28 and a maternal age within the range of 25 to 35, which is beneficial for eliminating the influence of BMI and year of pregnant woman on preterm delivery. In addition, the body weight of preterm infants was significantly lower than that of term infants, because of the infant development is not yet complete, which is in agreement with the result of this study (Kozuki et al., 2015).

The vaginal microbiota accounts for approximately 9% of the total human microbiota, which plays an essential role in improving vaginal health (Saraf et al., 2021). As a dynamic ecosystem composed of various microorganisms in different quantities and ratios, it helps preserve the integrity of the vaginal barrier and inhibits the growth of harmful bacteria (Shen et al., 2016). Recently, it has been reported that the composition of the vaginal microbiota often determines pregnancy outcomes (Salinas et al., 2020). In reproductive-age women, the proportion of *Lactobacillus* was obviously higher than that in others (Hayashida et al., 2022). Feehily et al. found vaginal *Lactobacillus* is consisted of *L. crispatus*, *L. delbruecki*, *L. gasseri*, *L. gasseriA*, *L. H fermentum*, *L. H gastricus*, *L. helveticus*, *L. jensenii*, *L. kefiranofaciens* and *L. taiwanensis* (Feehily et al., 2020). *Lactobacillus* is regarded as a probiotic that is beneficial for the host’s health when given in adequate amounts. *Lactobacillus* processes a series of physiological effects, such as suppressing oxidative stress and inflammatory responses (Qin et al., 2024). *Lactobacillus* also prevents the growth of harmful bacteria in the vagina by elevating the levels of short-chain fatty acids (Tachedjian et al., 2017). However, *Gardnerella* is a keystone genus in the vaginal microbiome, which has been confirmed to negative health outcomes including preterm birth (Berman et al., 2024). *Gardnerella* could destroy the vagina barrier and stimulate inflammatory response by degrading the protective mucins and up-regulating the NF-κB pathway, respectively (Schellenberg et al., 2016; Liu et al., 2024). Therefore, the lower abundance of *Lactobacillus* and high abundance of *Gardnerella* may be related to preterm delivery.

*Prevotella* could regulate mucin metabolism by stimulating both its production and degradation, which maintain the integrity of the vaginal barrier (Tett et al., 2021). *Trichococcus* functions as a short-chain fatty acid-producing bacteria, providing energy for the proliferation of vaginal epithelial cells (Xiang et al., 2022). *Gemella* is an essential member of the human microbiome in healthy individuals, and rarely causes systemic illness (Zaidi et al., 2018). In this study, the proportion of *Prevotella*, *Trichococcus*, *Neisseria* and *Gemella* was remarkably reduced in the PrPG group, while *Thauera*, *Ochrobactrum*, *Gardnerella*, *Massilia*, *Phyllobacteriaceae* and *Atopobium* were significantly increased. Among them, *Thauera* has the capacity to break down androgen under both aerobic and anaerobic conditions (Hsiao et al., 2023). *Ochrobactrum* is a non-enteric and Gram-negative organism, and its abundance is strongly related to inflammatory responses (Gigi et al., 2017). *Gardnerella* is widely distributed in women of childbearing age, and the high proportion of *Gardnerella* cause various diseases, which is extensively used to establish the bacterial vaginitis in animal (Wong et al., 2022; Jothi et al., 2024). *Massilia* belongs to the family Oxalobacteraceae and is associated with bacteremia, central nervous system infections, wound infections, lymphadenitis, and osteomyelitis (Ali et al., 2022). *Phyllobacteriaceae* has been confirmed to destroy carbohydrate and/or fat metabolism, as well as stimulate inflammatory responses by producing lipopolysaccharide (Chen et al., 2021). A high abundance of *Staphylococcus* can cause severe infectious diseases, such as impetigo, folliculitis, and cutaneous abscesses (Tabiś et al., 2022). The presence of *Staphylococcus* can promote the occurrence of inflammatory responses, which elevates the risk of preterm birth (Astley et al., 2019; Cappelletti et al., 2016). In addition, a previous study found that high abundance of *Atopobium* was positively associated with the incidence rate of infertility, endometritis, and pelvic inflammatory disease (Ravel et al., 2021). These results suggested that vaginal microbiota plays a vital role in altering the gestational age.

Apart from vaginal microbiota, vaginal metabolites play the most important role in pregnant women who later experienced preterm delivery (Kindschuh et al., 2023). 2-Piperidone could suppress the accumulation of reactive oxygen species and lipid peroxidation by regulating the activity of cytochrome P450 2E1 (Cheng et al., 2013). Obatoclax, a synthetic derivative of bacterial prodiginines, promotes the apoptosis of human colorectal carcinoma cells by suppressing Wnt/β-catenin signaling. Eurostoside acts as a useful organic compound that has been proven to inhibit the inflammatory responses by regulating the expression of inducible nitric oxide synthase (iNOS) and cyclooxygenase-2 (COX-2) (Le et al., 2023). Pregnanediol 3-O-glucuronide is a natural metabolite, and its levels are negatively associated with the risk of preterm delivery (Zhang et al., 2020). O-Phospho-L-serine acts as an inhibitor of serine racemase that can elevate the regulatory cytokine [(transforming growth factor-β (TGF‐β)] level and reduce the pro-inflammatory cytokines [tumor necrosis factor (TNF‐α) and interleukin-12p70 (IL-12p70)] in bone‐marrow‐derived dendritic cells (Fathallah et al., 2014). 1-Kestose is the smallest fructooligosaccharide component that modulates the composition of vaginal microbiota, particularly by up-regulating the abundance of *Bifidobacteria*, which protect the vaginal barrier (Tochio et al., 2018). Oral administration of 1-Kestose improves the symptoms of type 2 diabetes by elevating the short-chain fatty acids levels, such as acetate, butyrate and lactate (Watanabe et al., 2020). Short-chain fatty acids provide energy for vaginal epithelial cells, which is beneficial for the integrity of vaginal barrier. N-arachidonylglycine, an amino acid derivative of arachidonic acid, reduces CD4T cell responsiveness by reducing Th1 and Th17 cytokine levels, and regulating GPR18 MTORC1 signaling (Meadows et al., 2023). In this study, the relative contents of 2-Piperidone, Melphalan, N-acetylputrescine, Obatoclax, Eurostoside, Pregnanediol 3-O-glucuronide, O-Phospho-L-serine, 1-Kestose and N-arachidonylglycine in the PrPG group were obviously lower than that in the PrTG group. Acenocoumarol, a major organic compound in 4-hydroxycoumarins, is widely regarded as a potentially toxic compound (Valdivielso et al., 2004). Isopyrazam is a broad-spectrum succinate dehydrogenase inhibitor fungicide and has been confirmed to destroy the heart function by stimulating oxidative stress (Yan et al., 2023). Pentosidine is one of the best-characterized advanced glycation end-products that plays a pathological role in several aging-related disorders and can promote the development of diabetes (Li and Yu, 2018). Long-term consumption of hexose disrupt glucose metabolism, thereby elevating the risk of hyperglycemia and hyperlipidemia (Ai et al., 2023). 7-Hydroxymitragynine is widely used as an anesthetic, but its excessive use can result in a series of adverse reactions (Vento et al., 2021). A previous study found that high-fat diet accelerates the accumulation of phosphoethanolamine (PE) in the liver, which promotes liver function injury (Guo et al., 2020). Tamoxifen is considered a path-breaking medication in tumor treatment, but it has also been reported to give rise to thrombosis, epigastric discomfort and nausea (Wibowo et al., 2016). 1-Deoxynojirimycin destroys endoplasmic reticulum function, leading to the accumulation of unfolded or misfolded proteins (Lu et al., 2006). In the present study, Acenocoumarol, Isopyrazam, Pentosidine, hexose, 7-Hydroxymitragynine, PE, Tamoxifen and 1-Deoxynojirimycin concentrations in the PrPG group were higher than that in the PrTG group. Therefore, we deduce that the alterations in vaginal metabolites may be one of the important causes for preterm delivery.

## 5 Conclusion

In this study, we found a significant difference in vaginal microbiota between the PrPG and PrTG groups, characterized by reduced *Lactobacillus* and increased *Gardnerella*. Vaginal metabolomics analysis also revealed specific metabolites in pregnant women who later experienced preterm delivery, such as pregnanediol 3-O-glucuronide. These results offer useful information to enhance the accuracy of the model of premature birth prediction by integrating vaginal microbiomics and metabolomics.

## Data Availability

The data presented in the study are deposited in the NCBI database, accession number PRJNA1122359.
