# Supplementary material for: Insights into the alteration of vaginal microbiota and metabolites in pregnant woman with preterm delivery: prospective cohort study
Source: Front Cell Infect Microbiol. 2025 Sep 18;15:1580801. doi: 10.3389/fcimb.2025.1580801 (PMC12488658; doi:10.3389/fcimb.2025.1580801)

**Supplementary data**

**Table S1** The relative abundance of vaginal microbiota at the genus level between pregnant women who later experienced preterm delivery and term birth before cervical cerclage.

| Alteration | Key bacteria | PrPG/PrTG  (Fold change) |
| --- | --- | --- |
| Up-regulation | *Pseudoxanthomonas* | 11.11 |
|  | *Thauera* | 5.88 |
|  | *Ochrobactrum* | ∞ |
|  | *Olivibacter* | ∞ |
|  | *Gardnerella* | 100 |
|  | *Massilia* | 100 |
|  | *Phyllobacteriaceae_unclassified* | ∞ |
|  | *Buchnera* | ∞ |
|  | *Staphylococcus* | 5 |
|  | *Atopobium* | 50 |
| Down-regulation | *Prevotella 7* | 0.02 |
|  | *Trichococcus* | 0.01 |
|  | *Actinomyces* | 0.09 |
|  | *Neisseria* | 0.17 |
|  | *Lactobacillus* | 0.62 |
|  | *Rothia* | 0.04 |
|  | *Gemella* | 0.29 |
|  | *Haemophilus* | 0.23 |
|  | *Porphyromonas* | 0.08 |

**Table S2** The alteration in vaginal metabolites in pregnant women who later experienced preterm delivery in the positive ion modes.

| Alteration | Key metabolites | PrPG/PrTG  (Fold change) | |
| --- | --- | --- | --- |
| Down-regulation | Sarcosine ethyl ester | | 0.17 |
|  | Genistein | | 0.21 |
|  | PI(20:4(5Z,8Z,11Z,14Z)/0:0) | | 0.24 |
|  | N,N-Bis(2-hydroxyethyl)glycine | | 0.26 |
|  | N-Acetyl-S-farnesyl-L-cysteine | | 0.28 |
|  | 1-Deoxymannojirimycin (hydrochloride) | | 0.29 |
|  | Tamoxifen | | 0.30 |
|  | Desferrichrome | | 0.34 |
|  | Rhodamine 6G cation | | 0.36 |
|  | Isopetasoside | | 0.37 |
|  | PE(22:6(4Z,7Z,10Z,13Z,16Z,19Z)/0:0) | | 0.37 |
|  | Estriol-17-glucuronide | | 0.38 |
|  | 7-Hydroxymitragynine | | 0.39 |
|  | Buxifoliadine H | | 0.39 |
|  | Arg-Asn | | 0.40 |
|  | Hexose | | 0.42 |
|  | Marimastat | | 0.42 |
|  | Bipindogulomethyloside | | 0.43 |
|  | 3-Isoxazolecarboxamide | | 0.43 |
|  | Epoxyfumitremorgin_C | | 0.43 |
|  | 1-Phenylicosane-1,3-dione | | 0.43 |
|  | Pentosidine | | 0.44 |
|  | Pravastatin lactone | | 0.46 |
|  | Isopyrazam | | 0.47 |
|  | Gly-Pro-Arg | | 0.47 |
|  | Iprovalicarb | | 0.47 |
|  | Finasteridecarboxylic acid | | 0.49 |
|  | Caryoptosidic_acid | | 0.49 |
|  | Acenocoumarol | | 0.49 |
|  | Racemoramide | | 0.50 |
|  | 1,5-Naphthalenediamine | | 0.50 |
|  | Pimonidazole | | 0.50 |
| Up-regulation | N-Acetylputrescine | | 2.08 |
|  | 2-Piperidone | | 2.13 |
|  | Tetrahydrofurfuryl_acetate | | 2.13 |
|  | 3-Piperidinecarboxamide | | 2.23 |
|  | Merphalan | | 2.50 |
|  | Trimethoprim | | 2.78 |
|  | 1-[4-(4-Quinazolinylamino)phenyl]ethanone | | 2.94 |
|  | Tebuconazole | | 3.45 |
|  | N1,N1-Diethyl-1,6-hexanediamine | | 3.57 |
|  | 2,4,6-Trimethylpyridine | | 4.35 |

**Table S3** The alteration in vaginal metabolites in pregnant women who later experienced preterm delivery in the negative ion modes.

| Alteration | Key metabolites | PrPG/PrTG  (Fold change) |
| --- | --- | --- |
| Harmful metabolites | 2-Hydroxy-3-methylbutyric acid | 5.89 |
|  | 1,7-Bis(4-hydroxyphenyl)-4-hepten-3-one | 3.03 |
|  | Platyphylloside | 2.86 |
|  | Piroxicam | 2.70 |
|  | Oxypurinol | 2.56 |
|  | Xanthine | 2.56 |
|  | 1,6-Bis-O-(4-hydroxycinnamoyl)glucose | 2.56 |
|  | 6'-Sialyllactose | 2.27 |
|  | 9-Oxo-10(E),12(E)-octadecadienoic acid | 2.22 |
|  | Calceolarioside B | 2.22 |
|  | 9-HPODE | 2.17 |
| Beneficial metabolites | Lys-Val | 0.50 |
|  | Obatoclax | 0.50 |
|  | L-Alanyl-gamma-D-glutamyl-L-lysine | 0.50 |
|  | Ser-Ile | 0.49 |
|  | L-Alanyl-L-leucine | 0.47 |
|  | Val-Ile | 0.46 |
|  | Leu-Val | 0.46 |
|  | Lys-Gln | 0.46 |
|  | Eurostoside | 0.46 |
|  | 2'-N-Acetylparomamine | 0.45 |
|  | Pregnanediol 3-O-glucuronide | 0.44 |
|  | Ritalinic_acid | 0.44 |
|  | 1-Hydroxy-2-naphthoic acid | 0.43 |
|  | Leucylphenylalanine | 0.43 |
|  | Phe-Leu | 0.43 |
|  | N-Acryloyl-DL-aspartic acid | 0.41 |
|  | O-Phospho-L-serine | 0.41 |
|  | 3-(2-Chlorophenyl)-1H-pyrazol-5-amine | 0.38 |
|  | 16-Glucuronide-estriol | 0.37 |
|  | 2,2',4,4'-Tetrahydroxybenzophenone | 0.36 |
|  | Fluvoxamine_acid | 0.35 |
|  | 1-Kestose | 0.35 |
|  | Indoxyl sulfate | 0.35 |
|  | 2-Propenoic acid | 0.32 |
|  | His-Val | 0.32 |
|  | 2,4-Dodecadienamide | 0.31 |
|  | N-Arachidonylglycine | 0.28 |
|  | (3.beta)-Allopregnanolone sulfate | 0.25 |
|  | Phenylsulfate | 0.23 |
|  | 3-Phosphonopropanoic acid | 0.21 |
|  | 8-Acetyl-7-hydroxy-4-methylcoumarin | 0.18 |
|  | Mevalonic acid 5-pyrophosphate | 0.18 |

**Figure S1** Venn diagram Venn diagram illustrating the overlap of bacterial OTUs between the PrPG and PrTG groups.


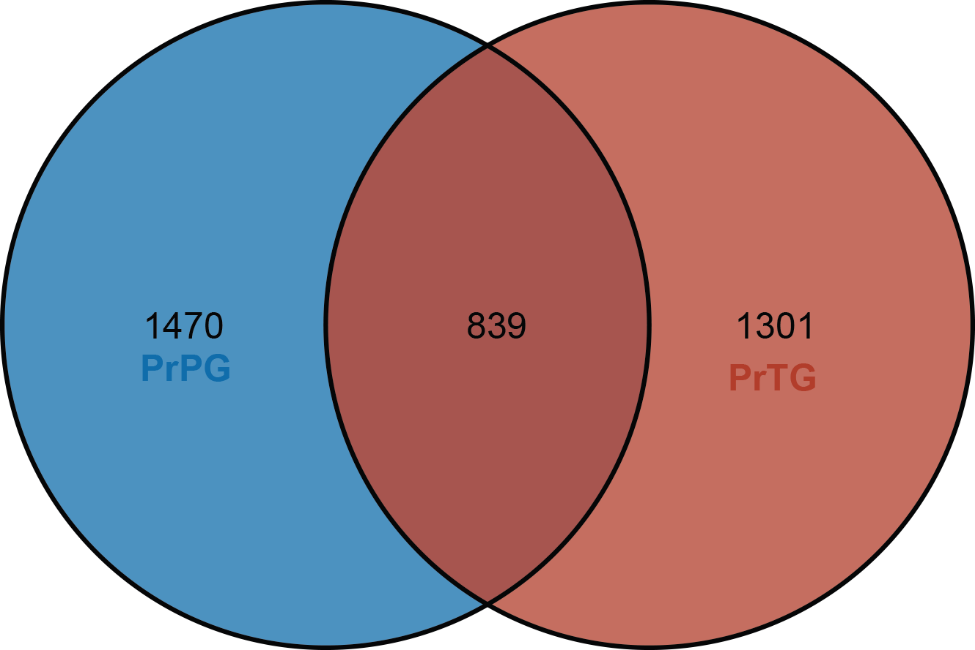

Supplement: Supplementary file 1 [file Table1.docx]
