# Supplementary figures and images for: Insights into the alteration of vaginal microbiota and metabolites in pregnant woman with preterm delivery: prospective cohort study
Source: Front Cell Infect Microbiol. 2025 Sep 18;15:1580801. doi: 10.3389/fcimb.2025.1580801 (PMC12488658; doi:10.3389/fcimb.2025.1580801)

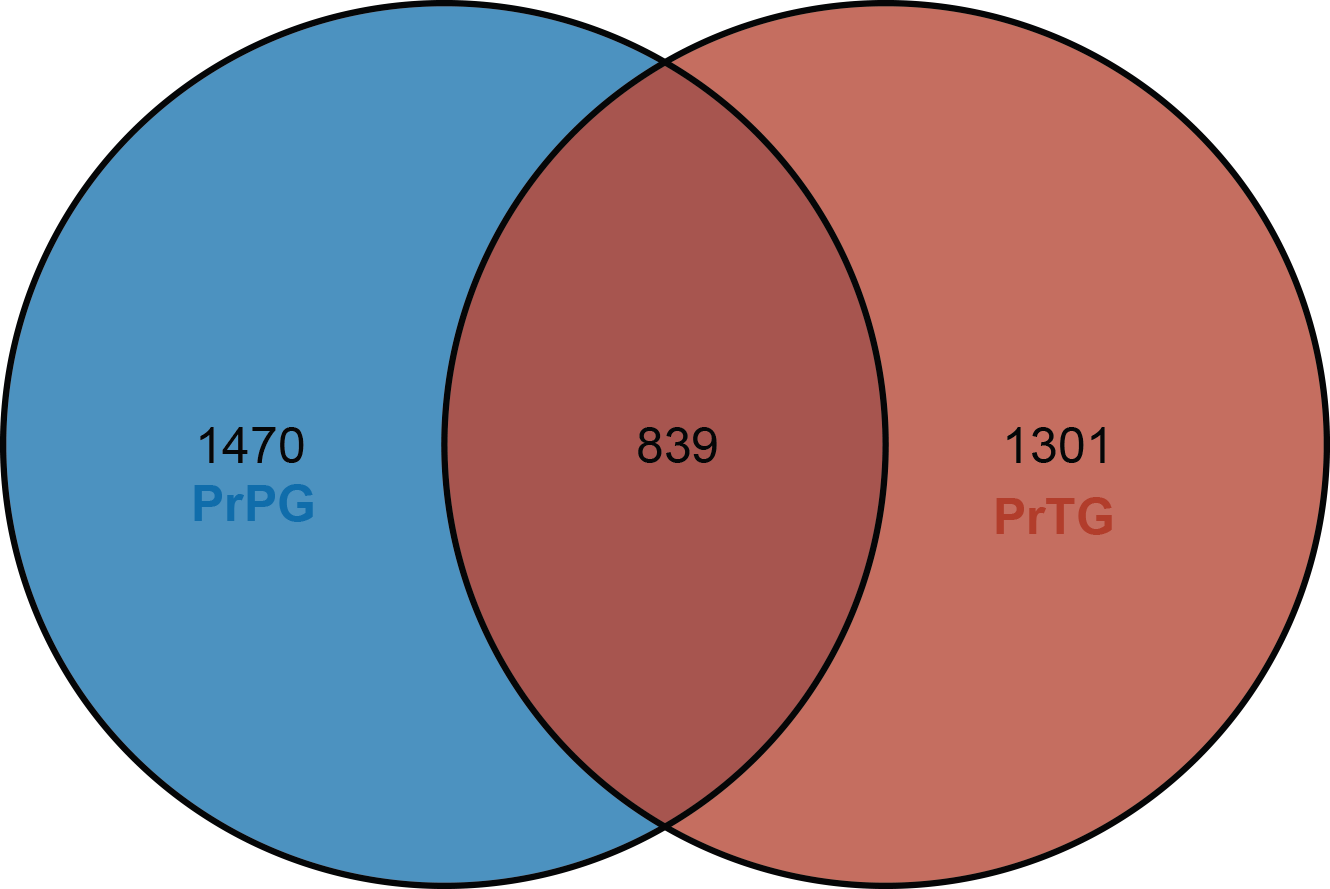

Supplement: Supplementary file 2 [file Image1.tif]
